# Robotic radical prostatectomy: difficult to start, fast to improve? Influence of surgical experience in robotic and open radical prostatectomy

**DOI:** 10.1007/s00345-021-03763-w

**Published:** 2021-07-16

**Authors:** Martin Baunacke, Awab Azawia, Johannes Huber, Christer Groeben, Christian Thomas, Angelika Borkowetz

**Affiliations:** Department of Urology, Medical Faculty Carl Gustav Carus, TU Dresden, Fetscherstr. 74, 01307 Dresden, Germany

**Keywords:** Surgical experience, Prostatectomy, Robotic, Prostate cancer, Positive surgical margin, Lymph node yield

## Abstract

**Purpose:**

The assistance of robotic systems raises the concern of whether there is an improved learning in robotic-assisted radical prostatectomy (RARP) compared to open retropubic radical prostatectomy (ORP).

**Methods:**

We retrospectively analyzed data from 1438 patients who underwent ORP (*n* = 735) or RARP (*n* = 703). For each procedure, the level of experience of three different surgeons was summarized. Perioperative and pathological parameters reflecting surgical performance were compared between both learning curves. RARP data were influenced by new introduction of the robotic system.

**Results:**

The median patient age at surgery was 66 years (IQR 42–80). Patients in the RARP group were younger (*p* < 0.001) and had a lower oncological risk (*p* < 0.001). Inexperienced RARP surgeons had a higher pT2-PSM rate and lower lymph node yield (13.8 ± 4.7 vs. 14.7 ± 4.8; *p* = 0.03) than inexperienced ORP surgeons. After 100 procedures, RARP and ORP surgeons had the same pT2-PSM rate (8% vs. 8%; *p* = 0.8) and lymph node yield (15.4 ± 5.4 vs. 15.4 ± 5.1; *p* = 1.0). In multivariate analysis for ORP, surgical inexperience (≤ 100 cases) was an independent predictor of a longer operating time (OR 9.0; *p* < 0.001) and higher amount of blood loss (OR 2.9; *p* < 0.001). For RARP, surgical inexperience (≤ 100 cases) was a predictor of a longer operating time (OR 3.9; *p* < 0.001), higher amount of blood loss (OR 1.9; *p* = 0.004), higher pT2-PSM rate (OR 1.6; *p* = 0.03), and lower lymph node yield (OR 0.6; *p* = 0.001).

**Conclusions:**

Surgical experience has a relevant impact on perioperative and pathological parameters RARP has a higher initial pT2-PSM rate and lower lymph node yield than ORP. This is relevant for patient selection for novice teaching in RARP.

**Supplementary Information:**

The online version contains supplementary material available at 10.1007/s00345-021-03763-w.

## Introduction

Radical prostatectomy (RP) is a curative treatment option for men with localized intermediate- and high-risk prostate cancer. Open retropubic radical prostatectomy (ORP) is the longest established procedure for RP. In addition to ORP, robotic-assisted radical prostatectomy (RARP) has been widely utilized since its introduction in 2000 [1]. Studies have shown improvements in surgical outcomes concerning complication rates, blood loss, transfusion rates and hospital stay [2]. Nevertheless, there are numerous studies comparing the oncological and functional outcomes of ORP and RARP with no definite resulting superiority [2–4]. However, in addition to the high standardization and advancement of technology, there will always be a determinant of surgical experience [5]. Nevertheless, there is the need to train new surgeons. Therefore, there will always be patients undergoing RARP or ORP who are part of a novice surgeon’s learning curve. However, a robotic system is a specific and different surgical tool reserved for specialized centres and a fewer number of surgeons, while exposure to open surgery is a common place for all surgeons. Therefore, some experience in open surgery would be expected by any novice ORP surgeon. The way of teaching novice surgeons differs between open and robotic-assisted surgery. Open surgery allows an almost simultaneous and close action of novice and attending surgeons. In contrast, robotic surgery creates a physical barrier with the need to change position or switch consoles between novice and attending surgeons to perform surgery together in an early learning curve [6].

The aim of our study was to evaluate the influence of surgical experience on surgical and oncological outcomes by comparing RARP and ORP.

## Patients and methods

### Study design

This retrospective study included 1438 patients treated with ORP (*n* = 735) or RARP (*n* = 703) by five surgeons between 2007 and 2018 at the Department of Urology, TU Dresden, Germany. Two surgeons provided their learning curves for ORP (*n* = 175 and *n* = 260), another two surgeons for RARP (*n* = 286 and *n* = 219) and one surgeon for ORP (*n* = 300) and RARP (*n* = 198). All surgeons who provided their RARP data had previous experience with at least 50 cases of ORP. All surgeons who provided their ORP data had previous experience in open tumor surgery. The objective for ORP novices lies in learning a new surgical procedure. The objective for RARP novices is learning a new technique. Our department had one of the first daVinci robotic systems in Germany. Data of one RARP surgeon are the initial experiences without a standardized teaching concept. He taught after his 50 procedures the two other RARP surgeons. The Department of Urology, TU Dresden, Germany, is a certified prostate cancer center. In our institution, experienced surgeons assisted novice surgeons during their first 50 radical prostatectomies. Therefore, surgeons performing the first 100 procedures, which include the first 50 supervised and the second 50 unsupervised procedures, were defined as inexperienced. Institutional review board approval was obtained for this study.

### Surgical details

All RARP procedures were performed transperitoneally using a 4-arm daVinci robotic system (Intuitive Surgical Inc., USA), initially using a single-console daVinci S robotic system. Since 2012, the daVinci Si system with an additional teaching console was used. The first 50 supervised procedures of all three RARP surgeons were before introduction of the additional teaching console. The assignment of patients to ORP or RARP was made by the surgeon depending on patient’s preference, comorbidity and aggressivity of cancer (according the d’Amico classification). The dissection of pelvic lymph nodes was performed in patients with intermediate- and high-risk prostate cancer and, depending on the patient’s preference, for those with low-risk cancer. The dissection of pelvic lymph nodes included external and internal iliac vessels with obturator fossa. In very few cases with preoperative evidence of extensive lymph node metastases, an extended lymphadenectomy may have been performed. However, we have no information on this. Nerve sparing RP was performed according to the tumor stage, preoperative erectile function and patient preference.

### Evaluated outcomes

The preoperative data comprised the baseline demographics, body mass index, ASA (American Society of Anesthesiologists) classification and D’Amico classification (D’Amico includes the PSA value, Gleason score and tumor stage). The operative parameters were intraoperative blood loss (content of surgical suction system minus rinsing), operating time (incision to closure time—including robot docking time), transfusion rate, complications, number of removed lymph nodes, prostate weight, overall positive surgical margin (PSM) and PSM only in the case of localized prostate cancer (≤ pT2). Complications included all grade III to V complications according to the Clavien–Dindo classification [7]. Missing data points are mentioned in tables.

### Statistics

Data were analyzed using the Chi^2^ test and *t* test. Binary logistic regression models were used for the multivariate estimation of risks and to predict outcome events. We used median of continues variables (prostate weight, operating time, blood loss, number of lymph nodes) for grouping in binary logistic regression models. *P* < 0.05 was considered to indicate significance. All calculations were performed with IBM SPSS Statistics 25.0 (Armonk, New York, USA).

## Results

### Collective

The median patient age at the time of surgery was 66 years (IQR 42–80). Patients who were treated by surgeons with more experience (> 100 RP) had a higher BMI (27.7 ± 4.1 vs. 27.0 ± 3.2, *p* < 0.001), a higher ASA score (ASA 3: 19% vs. 14%, *p* = 0.03) and a higher oncological risk (high risk: 24% vs. 22%, *p* < 0.001) (Suppl. Table 1). Patients with RARP were younger (63.4 ± 7.1 vs. 66.4 ± 6.4 years, *p* < 0.001), had a lower BMI (27.2 ± 3.7 vs. 27.6 ± 3.8 kg/m^2^, *p* = 0.03) and ASA score (ASA 3: 11% vs. 23%, *p* < 0.001), had a smaller prostate (51.5 ± 18.5 vs. 55.8 ± 22.9 g, *p* < 0.001) and had a lower oncological risk (high risk: 10% vs. 35%, *p* < 0.001) (Suppl. Table 1).

### Surgical performance

Patients who underwent ORP by inexperienced surgeons (≤ 100 ORP) had a longer operating time (163.9 ± 27.8 vs. 136.1 ± 24.8 min, *p* < 0.001), a higher amount of blood loss (1090.3 ± 511.8 vs. 908.7 ± 542.4 ml, *p* < 0.001), and a lower complication rate (3% vs. 7%, *p* = 0.02) and needed blood transfusion more often (12% vs. 6%, *p* < 0.001) than patients who underwent ORP by experienced surgeons (> 100 ORP) (Suppl. Tables 3, 4).

Patients who underwent RARP by inexperienced surgeons (≤ 100 RARP) had a longer operating time (233.7 ± 71.4 vs. 184.1 ± 40.0 min, *p* < 0.001), a higher amount of blood loss (888.4 ± 728.6 vs. 604.2 ± 609.4 ml, *p* < 0.001) and needed blood transfusion more often (9% vs. 2%, *p* < 0.001). Inexperienced surgeons removed fewer lymph nodes during lymphadenectomy (13.8 ± 4.7 vs. 15.4 ± 5.4, *p* < 0.001), had a higher total PSM rate (21% vs. 15%, *p* = 0.05) and pT2-PSM rate (PSM rate in pT2 tumors) (15% vs. 8%, *p* = 0.003) than experienced RARP surgeons (> 100 RARP) (Suppl. Table 5).

**Fig. 1:**
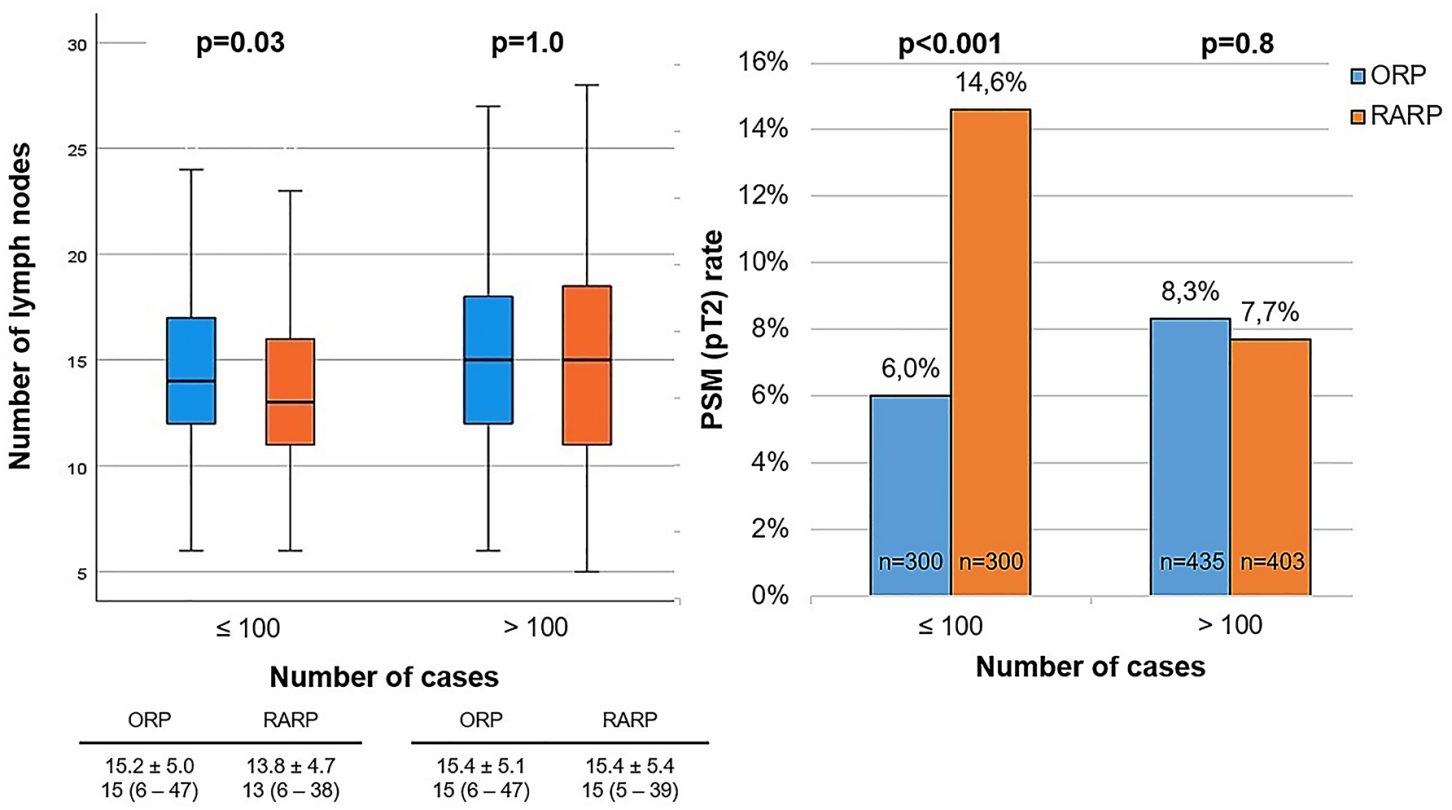
Lymph node yield and PSM rate of pT2 tumors depending on surgical experience

Inexperienced RARP surgeons had a higher pT2-PSM rate than inexperienced ORP surgeons (15% vs. 6%, *p* < 0.001) and a lower lymph node yield (13.8 ± 4.7 vs. 14.7 ± 4.8; *p* = 0.03) (Fig. 1). After 100 procedures, RARP and ORP surgeons had the same pT2-PSM rate (both 8%; *p* = 0.8) and the same lymph node yield (15.4 ± 5.4 vs. 15.4 ± 5.1; *p* = 1.0) (Fig. 1). There was a higher reduction in the mean operating time in RARP than in ORP from the initial 100 cases to > 100 cases (21.2% vs. 17.0%) and a higher reduction in the mean amount of blood loss from the initial 100 cases to > 100 cases (32.0% vs. 16.7%).

In multivariate logistic regression analysis, the independent predictors of a long operating time for ORP were obesity (≥ 30 kg/m^2^: OR 2.6; 9 < 0.001), additional lymph node dissection (OR 23.2; *p* < 0.001) and procedures performed by inexperienced surgeons (≤ 100 ORP: OR 9.0; *p* < 0.001). The independent predictors of a long operating time for RARP were additional lymph node dissection (OR 2.3; *p* = 0.008) and inexperienced surgeons (≤ 100 RARP: OR 3.9; *p* < 0.001) (Table 1).

**Table 1:** Comparison of multivariate analyses of predictors of surgical parameters between ORP and RARP

Long operating time	ORP (> 145 min)			RARP (> 196 min)		
	OR	95% CI	*p* value	OR	95% CI	*p* value
High ASA (2–3)	1.6	0.8–3.1	0.1	1.0	0.6–1.7	1.0
Older age (≥ 70 years)	1.0	0.7–1.4	0.8	0.9	0.6–1.4	0.6
Adiposities (BMI > 30)	2.6	1.7–3.9	** < 0.001**	1.5	0.9–2.3	0.09
Intermediate and high D'Amico	1.3	0.8–2.1	0.2	1.1	0.7–1.5	0.7
High prostate weight	1.2	0.8–1.7	0.3	1.2	0.9–1.7	0.2
With nerve sparing	0.8	0.5–1.1	0.1	0.6	0.4–1.1	0.09
With lymphadenectomy	23.2	5.1–104.5	** < 0.001**	2.3	1.2–4.2	**0.008**
≤ 100 procedures	9.0	6.2–13.1	** < 0.001**	3.9	2.7–5.7	** < 0.001**

High blood loss	ORP (> 900 ml)			RARP (> 500 ml)		
	OR	95% CI	*p* value	OR	95% CI	*p* value
High ASA (2–3)	1.5	0.8–2.8	0.2	0.6	0.3–1.0	0.06
Older age (≥ 70 years)	0.7	0.5–1.0	**0.04**	0.7	0.4–1.1	0.1
Adiposities (BMI > 30)	1.4	1.0–2.1	0.06	1.1	0.6–1.9	0.7
Intermediate and high D'Amico	1.8	1.2–2.7	**0.008**	1.1	0.7–1.7	0.7
High prostate weight	2.0	1.5–2.8	** < 0.001**	1.1	0.8–1.7	0.5
With nerve sparing	1.0	0.7–1.4	0.9	1.0	0.5–1.8	0.9
With lymphadenectomy	1.4	0.6–3.1	0.4	1.3	0.6–2.6	0.5
≤ 100 procedures	2.5	1.7–3.4	** < 0.001**	1.9	1.2–2.9	**0.004**

Complications (Clavien–Dindo ≥ 3)		ORP		RARP		
	OR	95% CI	*p* value	OR	95% CI	*p* value
High ASA (2–3)	1.7	0.4–7.2	0.5	0.6	0.2–1.5	0.2
Older age (≥ 70 years)	1.3	0.7–2.6	0.4	0.9	0.3–2.4	0.8
Adiposities (BMI > 30)	1.3	0.7–2.7	0.4	1.8	0.7–4.9	0.2
Intermediate and high D'Amico	1.8	0.7–5.0	0.2	1.3	0.6–3.2	0.5
High prostate weight	1.2	0.6–2.3	0.5	0.8	0.3–1.8	0.6
With nerve sparing	0.9	0.5–1.8	0.8	0.5	0.2–1.2	0.1
With lymphadenectomy	0.7	0.2–3.4	0.7	1.4	0.3–6.3	0.7
≤ 100 procedures	0.5	0.2–1.0	**0.04**	2.6	1.0–5.3	0.06

Positive surgical margins pT2		ORP		RARP		
	OR	95% CI	*p* value	OR	95% CI	*p* value
High ASA (2–3)	1.2	0.4–3.5	0.8	0.6	0.3–1.0	0.07
Older age (≥ 70 years)	0.6	0.3–1.1	0.1	1.5	0.9–2.4	0.1
Adiposities (BMI > 30)	1.1	0.6–2.1	0.8	2.6	1.6–4.4	** < 0.001**
Intermediate and high D'Amico	1.6	0.7–3.6	0.2	2.2	1.3–3.6	**0.002**
High prostate weight	0.5	0.3–1.0	**0.03**	0.8	0.5–1.2	0.3
With nerve sparing	1.1	0.6–2.0	0.7	1.2	0.6–2.2	0.6
With lymphadenectomy	2.3	0.3–17.6	0.4	0.8	0.4–1.6	0.5
≤ 100 procedures	0.7	0.4–1.3	0.3	1.6	1.0–2.6	**0.03**

High number of lymph nodes		ORP (> 15 lymph nodes)		RARP (> 14 lymph nodes)		
	OR	95% CI	*p* value	OR	95% CI	*p* value
High ASA (2–3)	1.1	0.6–1.9	0.8	1.0	0.6–1.6	0.9
Older age (≥ 70 years)	0.8	0.6–1.2	0.3	0.6	0.4–1.0	**0.03**
Adiposities (BMI > 30)	1.4	1.0–2.0	0.07	1.3	0.8–1.9	0.3
Intermediate and high D'Amico	1.3	0.9–1.9	0.2	1.5	1.0–2.1	**0.03**
High prostate weight	1.1	0.8–1.5	0.6	1.1	0.8–1.6	0.5
With nerve sparing	1.3	0.9–1.8	0.1	0.9	0.5–1.4	0.6
≤ 100 procedures	0.9	0.7–1.2	0.5	0.6	0.4–0.8	**0.001**

Continuous parameters were dichotomized by the median. Significance is indicated by bold numbers

The independent predictors for a high amount of blood loss in ORP were an intermediate or high D’Amico score (OR 1.8; *p* = 0.008), a younger age (≥ 70 years: OR 0.7; *p* = 0.04), a high prostate weight (> 51 g: OR 2.0; *p* < 0.001) and inexperienced surgeons (≤ 100 ORP: OR 2.9; *p* < 0.001). The predictor for a high amount of blood loss in RARP was inexperienced surgeons (≤ 100 RARP: OR 1.9; *p* = 0.004) (Table 1).

There was only one predictor for complications in ORP: inexperienced surgeon (OR 0.5; *p* = 0.04) (Table 1).

A high pT2-PSM rate in ORP was associated with a lower prostate weight (≥ 51 g: OR 0.5; *p* = 0.03). In RARP, a high pT2-PSM rate was associated with an intermediate or high oncological risk (OR 2.2; *p* = 0.002), with obesity (OR 2.6; *p* < 0.001) and with inexperienced surgeons (≤ 100 RARP: OR 1.6; *p* = 0.03) (Table 1).

There were only predictors for a high number of lymph nodes in RARP, but not in ORP: age (≥ 70 years: OR 0.6; *p* = 0.03), oncological risk (intermediate/high: OR 1.5; *p* = 0.03) and experience (≤ 100 RARP: OR 0.6; *p* = 0.001) (Table 1).

## Discussion

This study analyzed the influence of surgical experience on perioperative and pathological parameters comparing surgical performance in RARP and ORP. In ORP, surgical inexperience was associated with a longer operating time (OR 9.0) and higher amount of blood loss (OR 2.5). In RARP, surgical inexperience was associated with a longer operating time (OR 3.9), higher amount of blood loss (OR 1.9), higher risk for pT2-PSM (OR 1.6) and lower lymph node yield (OR 0.6). Surgical inexperience had a higher impact on operating time and blood loss in ORP than in RARP (operating time OR 9.0 vs. 3.9, blood loss 2.5 vs. 1.9). Novice RARP surgeons started with a worse pT2-PSM rate (15% vs. 6%) and lymph node yield (13.8 ± 4.7 vs. 14.7 ± 4.8) than novice ORP surgeons but were able to reach the same performance after the first 100 procedures.

Consistent with our data, different studies have shown a decrease in operating time and blood loss with increased surgical experience in RARP [8, 9] and ORP [10, 11]. Studies have shown a significant difference in operating time and blood loss between ORP and RARP resulting from their different approaches [2, 12]. In our study, we evaluated the impact of the learning curve in both procedures, showing a higher impact of the learning curve regarding operating time (OR: ORP 9.0 vs. 3.9 RARP) and blood loss (OR: ORP 2.5 vs. 1.9 RARP) in ORP. RARP showed a better improvement in the mean operating time (RARP 21.2% vs. 17.0% ORP) and mean blood loss (RARP 32.0% vs. 16.7% ORP) from the initial 100 cases to > 100 cases. One reason might be that most RARP surgeons have an advantage due to their previous experience in ORP. However, several studies have shown no difference regarding learning curves between RARP surgeons with and without experience in open surgery [13, 14].

There seemed to be a tendency toward a higher complication rate in inexperienced RARP surgeons (OR 2.6, *p* = 0.06). Several studies showed a decreasing complication rate with increasing experience from 15 to 4.5%, respectively, from 9.3 to 5% [15, 16]. A U.S. database study showed a decrease of RARP complication rate (similar to Clavien–Dindo grade 3–5) from 11.75% in first 25 cases to 8.9% in fourth 25 cases [17]. In contrast, experienced ORP surgeons had a higher complication rate than inexperienced ORP surgeons. We assume a beneficial effect of attending surgeons and positive patient selection during the first 100 ORP procedures for inexperienced surgeons, resulting in lower complication rates than in later ORP procedures compared to RARP procedures.

Surgical experience was an independent predictor for pT2-PSM in RARP but not in ORP. In contrast to our data, several studies have shown a relevant influence of surgical experience on PSMs in ORP. One study showed an improvement during the first 500–750 ORP procedures [10] with an overall pT2-PSM rate of 14%. Another study showed a decrease in the overall PSM rate from 40% with 10 prior cases to 25% with 250 prior cases [18]. Our total PSM rate was 24% and pT2-PSM rate was 7%; therefore, these rates are better than those in the previously mentioned studies with less potential for improvement. Optimized ORP training and patient selection for novice surgeons may have led to a lack of an effect of experience on PSMs in ORP in our data. In contrast, there is an influence of surgical experience on PSMs in RARP. A single-surgeon study with 1500 RARP procedures showed a 6.2 times higher pT2-PSM rate for RARP than for ORP at the first RARP procedure and became lower after 108 RARPs [19]. Our data showed the same effect with a steady pT2-PSM rate for ORP and a high starting rate for RARP in contrast to ORP (first 100 cases: 15% vs. 6%) and a lower rate in a higher case number (> 100 cases: 8% vs. 8%). This may reflect a more challenging early learning curve but a faster improvement in RARP than in ORP.

There are no studies evaluating learning curves for lymph node dissection in ORP and only two studies evaluating learning curves for RARP. Both studies showed an increase in the number of removed lymph nodes depending on the number of RARP cases [20, 21]. Comparing RARP and ORP, our data showed the same effect as for the PSM rate. In ORP, there was only a small increase in the lymph node yield from a mean of 14.7 ± 4.8 in the initial 100 cases to 15.4 ± 5.1 in > 100 cases. In RARP, the initial 100 cases started with a lower level of 13.8 ± 4.7 lymph nodes and increased to 15.4 ± 5.4 in the > 100 cases. Finally, the lymph node yield was the same in ORP and RARP, supporting studies with the same results [22] in contrast to earlier studies reporting a significantly higher yield in ORP [23, 24].

There are certain limitations to this study. This was a retrospective single-center study showing six learning curves of five different surgeons, reflecting the individual training concepts for ORP and RARP in our institution. This includes intentional patient selection with regard to comorbidity and d’Amico classification depending on the surgeon’s experience. However, multivariate analysis performed separately for each approach showed an impact of the learning curve on surgical parameters independent from the selection criteria. Because of the retrospective character of the study, there are missing data points. There seemed to be a bias especially in blood loss. It is most likely that there was no documentation because of no relevant blood loss reflecting in a high number of missing data points in RARP (RARP 227 vs. 47 ORP).

Finally, good results in terms of PSMs and complication rates support our selection and training concept in favor of patients. Another source of bias was the different surgical experiences of ORP and RARP surgeons. RARP surgeons had previous experience in ORP, with at least 50 ORP procedures, and performed ORP in parallel to RARP. Nevertheless, our data showed more complications and a worse pT2-PSM rate and lymph node yield for inexperienced RARP surgeons than for inexperienced ORP surgeons. Two of the studies already mentioned showed no benefit of previous experience in ORP for the RARP learning curve [13, 14]. A study analyzing the acquisition of robotic-assisted surgical skills between medical students and surgeons showed no transferability of laparoscopic or open surgical skills to robotic-assisted surgery [6]. A relevant limitation is that RARP data reflect the introduction of the robotic system in our clinic including learning curve of one surgeon who started without an experienced supervising surgeon and who taught after his first 50 procedures two other RARP surgeons. Furthermore, these data do not include new teaching possibility with an additional teaching console, which was introduced after first 100 procedures of all RARP surgeons.

One concern that arises is why novice RARP surgeons start with worse results than novice ORP surgeons. A relevant factor may be the introduction of a completely new surgical technology for inexperienced RARP surgeons. In contrast, the inexperienced ORP surgeons already had experience in open surgery in general. Another aspect is the more limited teaching techniques in RARP. A recent study analyzed instructional techniques used in robotic teaching environments [25]. They showed usage of a different set of instructional approaches compared to those used in open and laparoscopic surgery. In robotic surgery, intervention by the attending surgeon is only possible in overtaking the console or switching instrument control in the case of a teaching console, but not to perform surgery at the same time. A study comparing teaching techniques between ORP and RARP would provide additional insight.

## Conclusions

Surgical experience has a relevant impact on perioperative and pathological parameters. Novice RARP surgeons have a worse pT2-PSM rate and lymph node yield than novice ORP surgeons. After 100 cases, both sets of surgeons reached the same results in terms of pathological parameters. RARP seems to be a procedure with a higher initial barrier to entry. This should be recognized in patient selection for novice teaching in RARP. There is a need for teaching techniques to improve this initial hurdle.

## Supplementary Information

Below is the link to the electronic supplementary material.

Supplementary file1 (DOCX 27 KB)

Supplementary file2 (DOCX 27 KB)

Supplementary file3 (DOCX 26 KB)

Supplementary file4 (DOCX 27 KB)

Supplementary file5 (DOCX 27 KB)
