# Supplementary material for: Robotic radical prostatectomy: difficult to start, fast to improve? Influence of surgical experience in robotic and open radical prostatectomy
Source: World J Urol. 2021 Jul 16;39(12):4311–7. doi: 10.1007/s00345-021-03763-w (PMC8602152; doi:10.1007/s00345-021-03763-w)
Supplement: Supplementary file 1 — Supplementary file1 (DOCX 27 KB) [file 345_2021_3763_MOESM1_ESM.docx]

| **Variable** | | **All**  **(n=1438)** | **ORP**  **(n=735)** | **RARP**  **(n=703)** | **p value** |
| --- | --- | --- | --- | --- | --- |
| Age (years) | | 64.9 ± 6.9 | 66.4 ± 6.4 | 63.4 ± 7.1 | **<0.001** |
| Body mass index (kg/m²)  (57 missing data points) | | 27.4 ± 3.7 | 27.6 ± 3.8 | 27.2 ± 3.7 | **0.03** |
| ASA classification (13 missing data points) | 1 | 161 (11%) | 60 (8%) | 101 (15%) | **<0.001** |
|  | 2 | 1019 (72%) | 501 (69%) | 518 (74%) |  |
|  | 3 | 245 (17%) | 167 (23%) | 78 (11%) |  |
| Prostate weight (g)  (87 missing data points) | | 53.8 ± 21.1 | 55.8 ± 22.9 | 51.5 ± 18.5 | **<0.001** |
| Oncological risk  (3 missing data points) | Low | 452 (32%) | 158 (22%) | 294 (42%) | **<0.001** |
|  | Intermediate | 651 (45%) | 315 (43%) | 336 (48%) |  |
|  | High | 332 (23%) | 260 (35%) | 72 (10%) |  |
| Nerve sparing | Yes | 935 (65%) | 323 (44%) | 612 (87%) | **<0.001** |
|  | No | 503 (35%) | 412 (56%) | 91 (13%) |  |
| Lymphadenectomy | Yes | 1330 (92%) | 703 (96%) | 627 (89%) | **<0.001** |
|  | No | 108 (8%) | 32 (4%) | 76 (11%) |  |

Supplementary Table 1: Patient and operative characteristics according to the surgical procedure.
