# Supplementary material for: Robotic radical prostatectomy: difficult to start, fast to improve? Influence of surgical experience in robotic and open radical prostatectomy
Source: World J Urol. 2021 Jul 16;39(12):4311–7. doi: 10.1007/s00345-021-03763-w (PMC8602152; doi:10.1007/s00345-021-03763-w)
Supplement: Supplementary file 2 — Supplementary file2 (DOCX 27 KB) [file 345_2021_3763_MOESM2_ESM.docx]

| **Variable** | | **All**  **(n=1438)** | **≤ 100 RP (n=600)** | **>100 RP (n=838)** | **p value** |
| --- | --- | --- | --- | --- | --- |
| Age (years) | | 64.9 ± 6.9 | 64.8 ± 6.6 | 65.0 ± 7.1 | 0.7 |
| Body mass index (kg/m²)  (57 missing data points) | | 27.4 ± 3.7 | 27.0 ± 3.2 | 27.7 ± 4.1 | **<0.001** |
| ASA classification (13 missing data points) | 1 | 161 (11%) | 76 (13%) | 85 (10%) | **0.03** |
|  | 2 | 1019 (72%) | 435 (73%) | 584 (71%) |  |
|  | 3 | 245 (17%) | 86 (14%) | 159 (19%) |  |
| Prostate weight (g)  (87 missing data points) | | 53.8 ± 21.1 | 53.6 ± 21.2 | 53.9 ± 21.0 | 0.8 |
| Oncological risk  (3 missing data points) | low | 452 (32%) | 228 (38%) | 224 (27%) | **<0.001** |
|  | intermediate | 651 (45%) | 239 (40%) | 412 (49%) |  |
|  | high | 332 (23%) | 132 (22%) | 200 (24%) |  |
| Nerve sparing | Yes | 935 (65%) | 363 (61%) | 572 (68%) | **0.002** |
|  | No | 503 (35%) | 237 (39%) | 266 (32%) |  |
| Lymphadenectomy | Yes | 1330 (92%) | 541 (90%) | 789 (94%) | **0.005** |
|  | No | 108 (8%) | 59 (10%) | 49 (6%) |  |

Supplementary Table 2: Patient and operative characteristics according to the surgical experience.
