# Supplementary material for: Robotic radical prostatectomy: difficult to start, fast to improve? Influence of surgical experience in robotic and open radical prostatectomy
Source: World J Urol. 2021 Jul 16;39(12):4311–7. doi: 10.1007/s00345-021-03763-w (PMC8602152; doi:10.1007/s00345-021-03763-w)
Supplement: Supplementary file 3 — Supplementary file3 (DOCX 26 KB) [file 345_2021_3763_MOESM3_ESM.docx]

| **Variable** | | **All**  **(n=1438)** | **ORP**  **(n=735)** | **RARP**  **(n=703)** | **p value** |
| --- | --- | --- | --- | --- | --- |
| Clavien Dindo classification | <3 | 1366 (95%) | 693 (94%) | 673 (95%) | 0.4 |
|  | 3 | 69 (4%) | 40 (5%) | 29 (4%) |  |
|  | 4 | 0 (0%) | 0 (0%) | 0 (0%) |  |
|  | 5 | 3 (1%) | 2 (1%) | 1 (1%) |  |

Supplementary Table 3: Grade III-V complications according to the Clavien-Dindo classification. 72 grade III complications in 69 patients: 57 lymphocele therapies, 2 ureteric catheter insertions, 5 laparotomies because of bowel injury or bleeding, 8 surgical interventions because of wound dehiscence. 3 grade V complications: one myocardial infarction and two pulmonary embolisms.
