# Supplementary material for: Robotic radical prostatectomy: difficult to start, fast to improve? Influence of surgical experience in robotic and open radical prostatectomy
Source: World J Urol. 2021 Jul 16;39(12):4311–7. doi: 10.1007/s00345-021-03763-w (PMC8602152; doi:10.1007/s00345-021-03763-w)
Supplement: Supplementary file 4 — Supplementary file4 (DOCX 27 KB) [file 345_2021_3763_MOESM4_ESM.docx]

| **Variable** | | **All**  **(n=735)** | **≤ 100 ORP (n=300)** | **> 100 ORP (n=435)** | **p value** |
| --- | --- | --- | --- | --- | --- |
| Operating time | | 147.4 ± 29.4 | 163.9 ± 27.8 | 136.1 ± 24.8 | **<0.001** |
| Blood loss  (47 missing data points) | | 983.6 ± 537.1 | 1090.3 ± 511.8 | 908.7 ± 542.4 | **<0.001** |
| Blood transfusion | Yes | 61 (8%) | 36 (12%) | 25 (6%) | **0.003** |
|  | No | 674 (92%) | 264 (88%) | 410 (94%) |  |
| Complications (Clavien-Dindo grades 3 - 5) | Yes | 42 (6%) | 10 (3%) | 32 (7%) | **0.02** |
|  | No | 693 (94%) | 290 (97%) | 403 (93%) |  |
| Positive surgical margin | Yes | 177 (24%) | 66 (22%) | 111 (26%) | 0.3 |
|  | No | 558 (66%) | 234 (78%) | 324 (64%) |  |
| Positive surgical margin (T1/2) | Yes | 54 (7%) | 18 (6%) | 36 (8%) | 0.2 |
|  | No | 681 (93%) | 282 (94%) | 399 (92%) |  |
| Lymph nodes  (n=862) | | 15.2 ± 5.0 | 14.7 ± 4.8 | 15.4 ± 5.1 | 0.06 |

Supplementary Table 4: Surgical parameters according to the surgical experience in ORP.
