# Supplementary material for: Robotic radical prostatectomy: difficult to start, fast to improve? Influence of surgical experience in robotic and open radical prostatectomy
Source: World J Urol. 2021 Jul 16;39(12):4311–7. doi: 10.1007/s00345-021-03763-w (PMC8602152; doi:10.1007/s00345-021-03763-w)
Supplement: Supplementary file 5 — Supplementary file5 (DOCX 27 KB) [file 345_2021_3763_MOESM5_ESM.docx]

| **Variable** | | **All**  **(n=703)** | **≤ 100 RARP (n=300)** | **> 100 RARP (n=403)** | **p value** |
| --- | --- | --- | --- | --- | --- |
| Operating time | | 205.3 ± 60.8 | 233.7 ± 71.4 | 184.1 ± 40.0 | **<0.001** |
| Blood loss  (227 missing data points) | | 726.0 ± 677.1 | 888.4 ± 728.6 | 604.2 ± 609.4 | **<0.001** |
| Blood transfusion | Yes | 36 (5%) | 26 (9%) | 10 (2%) | **<0.001** |
|  | No | 667 (95%) | 274 (91%) | 393 (98%) |  |
| Complications (Clavien-Dindo grades 3 - 5) | Yes | 30 (4%) | 16 (5%) | 14 (3%) | **0.2** |
|  | No | 673 (96%) | 284 (95%) | 389 (97%) |  |
| Positive surgical margin | Yes | 125 (18%) | 63 (21%) | 62 (15%) | **0.05** |
|  | No | 578 (82%) | 237 (79%) | 341 (85%) |  |
| Positive surgical margin (T1/2) | Yes | 75 (11%) | 44 (15%) | 31 (8%) | **0.003** |
|  | No | 628 (89%) | 256 (85%) | 372 (92%) |  |
| Lymph nodes  (n= 627) | | 14.8 ± 5.1 | 13.8 ± 4.7 | 15.4 ± 5.4 | **<0.001** |

Supplementary Table 5: Surgical parameters according to the surgical experience in RARP.
